# Purinergic P2 Receptors: Structure and Function 2.0

**DOI:** 10.3390/ijms24065462

**Published:** 2023-03-13

**Authors:** Hana Zemkova

**Affiliations:** Institute of Physiology, Czech Academy of Sciences, 14220 Prague, Czech Republic; hana.zemkova@fgu.cas.cz

This Special Issue of the *International Journal of Molecular Sciences (IJMS)* is a direct continuation of the previous Special Issue of this journal, entitled “Purinergic P2 Receptors: Structure and Function” https://www.mdpi.com/journal/ijms/special_issues/Purinergic_Receptors_2 (accessed on 31 May 2022). It contains seven original research papers written by experts in the field of purinergic signaling, who present recent advances in the investigations of P2X and P2Y receptors. These articles cover a range of topics, from molecular structure–function relationships to the physiology and pathophysiology of P2 receptors, and highlight the importance of these receptors as possible pharmacological targets for clinical applications.

## 1. Molecular Structure–Function Relationships of P2 Receptors

The P2X4 receptor is known to be allosterically modulated by zinc. Peralta et al. used molecular dynamic simulations to explain the nature of Zn^2+^ binding to the P2X4 wild-type and alanine substitution of cysteine residues in the head domain of the receptor. This computational analysis discovered new details about Zn^2+^ interactions with extracellular cysteines and showed that these interactions are based on physicochemical principles that are applicable to other proteins where Zn^2+^ plays a modulatory role [1]. In addition to being expressed at the plasma membrane, P2X4 receptors are also localized to endolysosome membranes. Lysosomal P2X4 may be activated by the high level of ATP that is accumulated in lysosomes and inhibited by the typically acidic pH within the lumen (pH~4.5). Tan et al. described the regulation of lysosomal P2X4 by two unrelated membrane receptors: the P2X7 and H1 histamine receptors. They used transfected normal rat kidney (NRK) cells and HeLa cells to show that increases in intracellular Ca^2+^ level triggered by activation of P2X7 or H1 receptors are amplified by lysosomal P2X4 receptors. This role of the P2X7 or H1 receptor is mediated by the regulation of lysosome acidity, and the resulting P2X4-dependent Ca^2+^ release from lysosomes causes anterograde trafficking, redistribution and fusion of lysosomes [2]. Dunning et al. tested a hypothesis that P2X7 current facilitation and large pore formation induced by prolonged agonist stimulation depend on the formation of functional complexes of the P2X7 and TMEM16 channels. Using whole-cell and single-channel electrophysiology, pharmacology, CRISPR/Cas9 technology and cell imaging, they showed that P2X7 is able to interact with endogenously expressed TMEM16 channels in both HEK293T cells and Xenopus oocytes; this coupling contributes to current facilitation and partially to YO-PRO-1 uptake, and the functional interaction of P2X7 and TMEM16 is retained in excised patches. Using single-channel patch clamp recording, they found that agonist-evoked current facilitation predominantly originates from a large increase of P2X7 open probability, and this effect is mimicked by the depletion of plasma membrane cholesterol and modulated by drugs such as flufenamic acid (FFA) and 9-anthracene-carboxylic acid (9-AC), which modulate the activity of TMEM16, including TMEM16F [3]. Dsouza et al. investigated the potency of the agonist of the P2Y13 receptor, the ADP-stimulated G-protein-coupled receptor implicated in many physiological processes, including neurotransmission, metabolism, pain and bone homeostasis. The authors conduced a meta-analysis of studies that investigated P2Y13 sensitivity to different agonists, compared EC50 values and Hill coefficients in different systems (transfected and native) and normalized the data. They found that ADP-like agonists are much more effective compared to ATP-like agonists and that ADP-like agonists are also more potent for human P2Y13 compared to rodent P2Y13, indicating that the P2Y13 appears to be the most ADP-sensitive receptor characterized to date [4].

## 2. Physiology of P2 Receptors

It is assumed that the activation of P2X7 at central and peripheral sites in trigeminal pain pathways contribute to an increase in ocular hyperalgesia and microglia activation in dry eye disease. Puro et al. investigated the role of P2X7 in both the physiology and the pathobiology of the goblet cells located in the conjunctiva. Using high-temporal resolution membrane capacitance measurements, they monitored the exocytotic activity of single goblet cells and found that activation of P2X7 boosts neural-evoked exocytosis and accelerates the replenishment of mucin-filled granules after exocytotic depletion. They conclude that P2X7 activation exerts a yin–yang effect on conjunctival goblet cells: the high-gain benefit of enhancing the supply of tear-stabilizing mucin is implemented at the high risk of endangering goblet cell survival [5]. Another study explored the anti-thrombogenic efficacy of blockade of the P2X7 in vascular system. Marchase et al. used a confocal blood flow videomicroscopy system on cryosections of internal mammary artery or carotid plaque to determine/localize platelets and fibrin in relation to functional expression of P2X7. They found that the sections from carotid plaque, but not internal mammary artery, pre-treated with the P2X7 antagonist A740003, demonstrated poor thrombogenesis in flow experiments, suggesting that P2X7 modulation might be possibly used for atherothrombosis prevention/therapy [6]. The study by Lommen et al. focused on the spatial and temporal distribution of various P2 receptors in the hippocampus, the brain region of fundamental importance for learning and memory. The authors performed an extensive and systematic analysis of circadian changes and distribution patterns of expression of P2X1-7, P2Y1,2, P2Y4, P2Y6 and P2Y11–14 immunoreaction in different layers of the hippocampus during the 24 h cycle. They showed that all fifteen P2 receptors, except P2X4 and P2Y1, were at least moderately expressed in any of the hippocampal layers and that the temporal distribution of P2 receptors can be segregated into two large time windows: early-to-mid-day and mid-to-late night. This study provides an important basis for understanding circadian rhythmicity of P2 purinergic signaling in the hippocampal glia/neuronal network and identified the best time window for modulating synaptic plasticity and drug efficacy by P2-mediated purinergic signaling [7].

## 3. Conclusions

This Special Issue shows that understanding the processes regulating the function and expression of ATP/ADP-activated P2 receptors is crucial for the development of new therapeutic strategies for a wide variety of cardiovascular, immune and neurodegenerative diseases, such as atherothrombosis, dry eye disease and Alzheimer’s disease.
